# The Impact of Prehabilitation on Post-operative Outcomes in Oesophageal Cancer Surgery: a Propensity Score Matched Comparison

**DOI:** 10.1007/s11605-020-04881-3

**Published:** 2020-12-02

**Authors:** Laura J. Halliday, Emre Doganay, Venetia A. Wynter-Blyth, George B. Hanna, Krishna Moorthy

**Affiliations:** 1 Department of Surgery and Cancer, Imperial College London, London, UK; 2 Oesophago-gastric cancer surgery unit, St Mary’s Hospital, Imperial College Healthcare NHS Trust, London, UK

**Keywords:** Oesophageal cancer, Exercise therapy, Preoperative care, Surgery

## Abstract

**Background:**

Patients undergoing oesophageal cancer surgery are often frail with a high risk of post-operative complications. Prehabilitation has been shown to reduce post-operative complications in specific patient populations but evidence in oesophageal cancer patients is inconclusive.

**Methods:**

Between January 2016 and April 2019, all patients with resectable oesophageal cancer who underwent curative treatment at a specialist tertiary centre participated in a personalised, home-based, multimodal prehabilitation programme. Post-operative complications and hospital stay in this group were compared to a control sample. Propensity score matching was used to control for differences in baseline characteristics.

**Results:**

Seventy-two patients who completed prehabilitation and 39 control patients were studied; following propensity score matching, there were 38 subjects in each group. In comparison to matched controls, patients in the prehabilitation group had a lower incidence of post-operative pneumonia (prehabilitation = 26%; control = 66%; *p* = 0.001) and a shorter length of stay (prehabilitation = median 10 days, IQR 8–17 days; control = median 13 days, IQR 11–20 days; *p* = 0.018). On multivariate regression analysis, participation in prehabilitation was associated with a 77% lower incidence of post-operative pneumonia (OR 0.23, 95% CI 0.09 to 0.55 *p* = 0.001). There was no significant difference in the incidence of overall complications or severe complications.

**Conclusion:**

Prehabilitation was associated with a lower incidence of post-operative pneumonia and shorter hospital length of stay following oesophagectomy. This model of home based, personalised, and supervised prehabilitation is effective and relevant to centralised cancer services.

## Introduction

Patients undergoing oesophageal cancer surgery are often elderly, frail, malnourished, with poor functional reserve, all of which are associated with poor postoperative outcomes.^1^–^4^ Up to 60% of patients experience post-operative complications.^5^ The high morbidity can lead to a prolonged hospital stay, delayed recovery, long-term disability, and poor survival.^6^–^8^.

*Prehabilitation* utilises the time before surgery to improve a patient’s functional capacity to better withstand the stress of surgery.^9^,^10^ Through activation of inflammatory, endocrine, and immunological responses, the ‘surgical stress response’ creates increased metabolic demand and a pro-catabolic state. 11 The introduction of neoadjuvant chemotherapy provides a survival benefit but at a cost to functional capacity when it is most needed; immediately prior to surgery.^12^–^14^ This physical deconditioning represents a clear mismatch with the metabolic demand being placed on the body during and after an oesophagectomy.

The content of prehabilitation programmes in the published literature varies considerably, from a one-off appointment at a ‘surgery school’ to a personalised exercise programme lasting for several weeks.^15^–^17^ There is growing recognition that in order to maximise the benefits that can be accrued in the pre-operative period, programmes should be multimodal, including exercise, nutritional support, and psychological support.^15^,^18^–^20^

Prehabilitation has been trialled in a range of surgical specialities, including colorectal, breast, hepatobiliary, bariatric, urological, thoracic, and orthopaedic surgery.^10^,^16^,^17^,^21^,^22^ There is a large body of evidence for prehabilitation in patients undergoing intra-abdominal surgery, with studies reporting improvements in pre-operative cardiopulmonary fitness,^14^,^17^,^23^,^24^ post-operative cardiopulmonary fitness,^23^–^26^ and reductions in post-operative complications.^22^,^27^

However, the evidence in oesophageal cancer is scarce.^20^ Surgery for oesophageal cancer has a high morbidity and is associated with a prolonged recovery^5^,^28^,^29^ so there is significant potential to improve outcomes in this high-risk population. However, there are few studies of prehabilitation in oesophageal cancer and to date, none has shown significant changes in post-operative complications.^30^,^31^

The aim of this study is to establish the effect of a personalised, home-based prehabilitation programme on post-operative outcomes following oesophageal cancer surgery.

## Materials and Methods

### Study Design

PREPARE (Physical activity, Respiratory exercises, Eat well, Psychological well-being, Ask about medications, Remove bad habits, Enhanced recovery) for Surgery is a personalised, home-based, multimodal prehabilitation programme for patients with oesophageal and gastro-oesophageal junctional cancer. All patients referred to the oesophago-gastric multi-disciplinary team, aged ≥ 18 years, and with a diagnosis of potentially resectable oesophageal cancer were invited to participate in the PREPARE for Surgery programme. Exclusion criteria included inability to give informed consent, non-resectable disease, and patients who declined surgery. All patients who completed the programme between January 2016 and March 2019 were included in this study.

A control group of patients who underwent resection at the same centre but had not undertaken prehabilitation was identified. This group comprised of patients who received treatment in 2015 (before the introduction of the programme), alongside patients from 2016 to 2018 who underwent resection at the same centre as the prehabilitation cohort but either started their treatment at a different centre so did not participate in the prehabilitation programme or were not included in the programme for administrative reasons. Ethical approval for analysis of patient data was granted by the UK Health Research Authority (268837) and the study protocol registered with Imperial College London (19SM5445).

### The PREPARE for Surgery Programme

Patients attended the multi-professional prehabilitation clinic within one week of the completion of staging investigations, before or just at the commencement of neoadjuvant chemotherapy. The patients were reviewed by a cancer clinical nurse specialist (CNS), exercise specialist, and dietitian during a single visit to the hospital to develop and agree to a personalised prehabilitation plan.

#### Exercise Programme

Details of our exercise programme have previously been published^32^ and are summarised below. A personalised, home-based exercise programme was prescribed by an exercise therapist. In keeping with WHO guidelines, patients were prescribed a minimum of 600 MET minutes week^−1^, which equates to 150 min of moderate intensity activity, with the aim of increasing this to 1200 MET minutes week^−1^ (300 min of moderate intensity activity).^33^

Patients were prescribed a mixture of aerobic and strength exercises, each with a defined frequency, intensity, and duration.^34^ The exercises were personalised according to the results of submaximal exercise testing, activities of daily living, previous exercise behaviour, patient preference, medical co-morbidities, and social circumstances. Patients received training on how to undertake the exercises and how to self-regulate the intensity using the Borg scale rating of perceived exertion (RPE),^35^–^37^ with a target range of 13 to 15 depending on the patient’s medical history and type of exercise.

A weekly telephone touch-point was held with an exercise therapist. Providing the goals were achieved, the exercise programme was progressed by frequency, duration, and then intensity. For those who were unable to meet their goals, the programme was adapted to their clinical condition and re-evaluated at the next touch-point. The method for assessing adherence to the exercise programme has previously been reported^32^ and was expressed as the volume of exercise completed by the patient each week in MET minutes week^−1^ divided by the prescribed volume of exercise each week in MET minutes week^−1^.

#### Nutritional Support

A specialist dietitian undertook an assessment of nutritional status including identification and stratification of nutritional risk. A plan was agreed based on symptoms, dietary eating habits, and nutritional deficiencies. Weekly or fortnightly phone calls from the dietitian were used to monitor adherence to the programme. Interventions, such as oral supplementation or enteral feeding via a jejunostomy, were established when risk was identified.

#### Psychological Support

The overall aim was to contain anxiety, facilitate adaptation to their current psychological health and disease state, and improve self-efficacy. Psychometric screening was completed immediately prior to the first CNS consultation to assess anxiety, depression, social support, self-efficacy, and relevant background information (such as past trauma, major life events, mental health history). The aims the consultation were threefold: (i) to explore and address any anxieties or concerns the patient may have regarding their diagnosis, symptoms, and/or treatment plan; (ii) to outline the rationale for prehabilitation and identify any potential barriers or facilitators to adherence; (iii) to facilitate positive behaviour change.

Motivational interviewing techniques were used to engage patients, establish their motivation for change, and increase their sense of confidence and control.^38^ This was accompanied by a timeline of agreed goals with personalised written and visual information.

### Study Outcomes

The primary study outcome was 60-day post-operative complications. Other outcomes included 60-day pulmonary complications, severe complications (Clavien Dindo grade 3 and higher), length of stay, and 30-day readmission rate. The 60-day end-point for complications was chosen to ensure that any complications that resulted in readmission within 30 days of discharge would be included in the overall complication figure. Complications were defined according to the Esophagectomy Complication Consensus Group (ECCG) guidelines.^39^

**Fig. 1:**
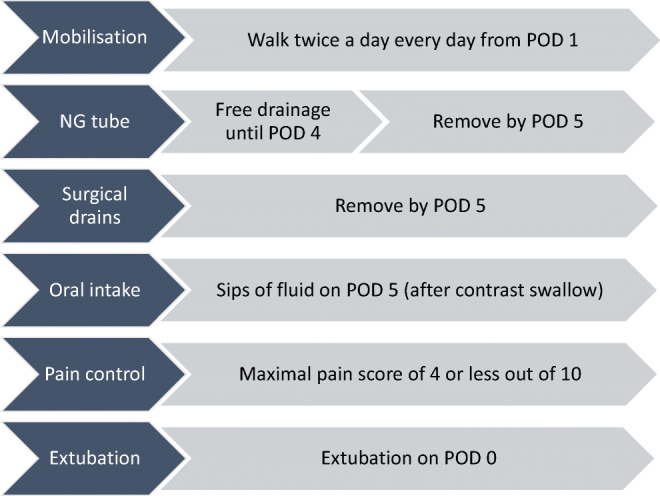
Enhanced recovery protocol following oesophagectomy. NG, nasogastric; POD, post-operative day

The unit has an Enhanced Recovery Programme (ERP) since 2014 and the same protocol was used for both cases and controls (Fig. 1). Compliance with each element of the ERP was measured to assess whether there were any differences in post-operative care and early recovery between the two groups.

### Statistical Analysis

To reduce the effects of potential confounding factors on the comparison between the two groups, a propensity score matched analysis was performed.^40^,^41^ A propensity score was created using a multivariate logistical regression model, with the following potential confounding factors as covariates: age, ASA grade, pre-operative cancer staging, use of neoadjuvant chemotherapy, and Charlson Comorbidity Index (CCI). Using the propensity score, patients in the prehabilitation group were matched 1:1 to those in the control group.

Outcomes were compared between cases and controls using chi-squared tests for categorical outcomes. For continuous outcomes, either Mann-Whitney or unpaired *T* tests were used, depending on distribution. Multivariate analysis of factors associated with complications was performed using binary logistic regression. Two-tailed tests were used throughout with a significance level of *p* < 0.05. Statistical analysis was performed using SPSS version 25 (IBM, New York, USA).

## Results

**Fig. 2:**
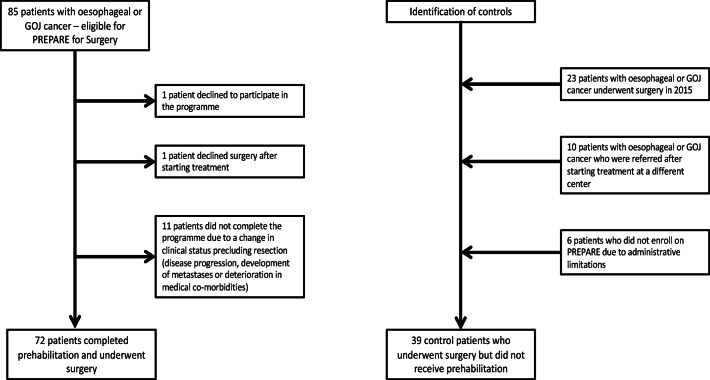
Study participant flow chart

Seventy-two patients who completed the programme between January 2016 and April 2019 and underwent surgery were included in this study (Fig. 2). Thirty-nine control patients who underwent surgery but did not complete prehabilitation were identified (Fig. 2).

Prior to propensity score matching, there were no statistically significant differences in age, ASA grade, clinical stage, CCI, the use of chemotherapy, operative approach, pre-operative weight, or pre-operative body mass index between the two groups (Table 1). After propensity score matching, 38 matched pairs were generated. There was a higher incidence of respiratory comorbidities in the PREPARE group in comparison to the controls, but there was no difference in the incidence of cardiac, endocrine, neurological, or psychological comorbidities (Table 1).

**Table 1:** Demographic factors before and after application of PS matching

	Unmatched groups			Matched groups		
	PREPARE	Controls	*p* value	PREPARE	Controls	*p* value
*N*	72	39		38	38	
Age—median (IQR)	68 (61–73)	67 (62–74)	0.973	69 (60–73)	68 (61–74)	0.979
ASA, *n* (%)			0.557			0.574
1	0	0		0	0	
2	59 (82%)	30 (77%)		31 (82%)	29 (76%)	
3	12 (17%)	9 (23%)		7 (18%)	9 (24%)	
4	1 (1%)	0		0	0	
Stage, *n* (%)			0.736			0.842
1	7 (10%)	4 (10%)		2 (5%)	4 (10%)	
2	8 (11%)	4 (10%)		5 (13%)	4 (10%)	
3	44 (61%)	21 (54%)		21 (55%)	21 (55%)	
4	13 (18%)	10 (26%)		10 (27%)	9 (27%)	
CCI—median (IQR)	5 (4–5)	5 (4–5)	0.872	5 (2–8)	5 (4–5)	0.944
NAC, *n* (%)	63 (88%)	21 (87%)	0.961	33 (87%)	33 (87%)	> 0.999
Pre-operative weight (kg)—mean (SD)	80.7 (18.6)	77.7 (13.9)	0.340	81.9 (18.9)	77.0 (12.6)	0.217
Pre-operative BMI (kg/m^2^)—mean (SD)	27.4 (5.8)	26.1 (3.7)	0.154	27.5 (5.6)	25.9 (3.7)	0.172
Comorbidities						
Cardiac	18 (25%)	5 (13%)	0.131	10 (26%)	5 (13%)	0.150
Respiratory	19 (26%)	2 (5%)	0.006	11 (29%)	2 (5%)	0.006
Neurological	5 (7%)	1 (3%)	0.330	1 (3%)	1 (3%)	> 0.999
Endocrine	11 (15%)	6 (15%)	0.988	4 (11%)	6 (16%)	0.497
Psychiatric	8 (11%)	5 (13%)	0.789	2 (5%)	5 (13%)	0.234
Procedure			0.152			0.280
Three stage	12 (17%)	11 (28%)		7 (18%)	11 (29%)	
Two stage	60 (83%)	28 (72%)		31 (82%)	27 (71%)	
Operative approach—abdominal stagea			0.450			0.692
Laparotomy	66 (92%)	34 (87%)		34 (89%)	35 (92%)	
Laparoscopy	6 (8%)	5 (13%)		4 (11%)	3 (8%)	

^a^In all cases, the thoracic component was performed as an open procedure

Continuous data displayed as mean (standard deviation) if parametric and median (interquartile range) if non-parametric. *CCI*, Charlson Comorbidity Index; *NAC*, neoadjuvant chemotherapy; *ASA*, American Society of Anesthesiologist physical status classification; *BMI*, body mass index; *IQR*, inter-quartile range; *SD*, standard deviation

In the PREPARE group, no patients asked to withdraw from the programme once they had enrolled. The mean amount of physical activity completed each week was 848 MET minutes week^−1^ (SD 659) during neoadjuvant chemotherapy, and 1198 MET minutes week^−1^ (SD 1225) after chemotherapy was completed. The mean adherence to the personalised exercise prescriptions was 55% (SD 29.8) during chemotherapy and 66% (SD 35.9) after it was completed.

### Post-operative Outcomes

The overall incidence of complications was lower in the PREPARE group compared to controls, but this difference was not statistically significant (Table 2; unmatched analysis 68% vs 79%, *p* = 0.089; matched analysis 63% vs 82%, *p* = 0.073).

**Table 2:** Comparison of study outcomes in both unmatched and propensity score matched analysis

	Unmatched groups			Matched groups		
	PREPARE	Controls	*p* value	PREPARE	Controls	*p* value
Any complication, *n* (%)	46 (68%)	31 (79%)	0.089	24 (63%)	31 (82%)	0.073
Pulmonary complication, *n* (%)	26 (36%)	26 (67%)	0.002	12 (32%)	26 (68%)	0.001
Post-operative pneumonia, *n* (%)	24 (33%)	25 (64%)	0.002	10 (26%)	25 (66%)	0.001
Severe complications, *n* (%)a	17 (24%)	18 (46%)	0.015	12 (32%)	18 (47%)	0.159
Length of stay (days), median (IQR)	10 (8–17)	13 (11–20)	0.019	10 (8–17)	13 (11–20)	0.018
30-day readmission, *n* (%)	13 (18%)	3 (8%)	0.138	9 (24%)	3 (8%)	0.059
Enhanced recovery protocol compliance						
Mobilisation, *n* (%)	24 (33%)	14 (36%)	0.679	11 (29%)	13 (34%)	0.449
NGT removal, *n* (%)	40 (56%)	13 (33%)	0.053	23 (61%)	13 (34%)	0.046
Drain removal, *n* (%)	34 (47%)	11 (28%)	0.048	16 (42%)	11 (29%)	0.179
Oral intake, *n* (%)	28 (39%)	12 (31%)	0.442	15 (39%)	12 (32%)	0.583
Fluid balance, *n* (%)	3 (4%)	4 (10%)	0.203	1 (3%)	4 (11%)	0.144
Pain control, *n* (%)	41 (57%)	23 (59%)	0.656	21 (55%)	23 (61%)	0.362
Day 0 extubation, *n* (%)	51 (71%)	28 (72%)	0.905	27 (71%)	27 (71%)	> 0.999

^a^Severe complications was defined as Clavien Dindo grade 3 or higher

*IQR*, inter quartile range; *NGT*, nasogastric tube

In both the unmatched and the matched analyses, there was a significant reduction in the incidence of 60-day pulmonary complications and post-operative pneumonia in the PREPARE patients in comparison to controls (Table 2; unmatched analysis overall pulmonary complications 36% vs 67% *p* = 0.002 and post-operative pneumonia 33% vs 64% *p* = 0.002; matched analysis overall pulmonary complications 32% vs 68% *p* = 0.001 and post-operative pneumonia 26% vs 66% *p* = 0.001).

There was a shorter length of stay in the PREPARE group in comparison to controls (10 days vs 13 days in both the unmatched and matched analyses, *p* = 0.019 and *p* = 0.018 respectively). In the unmatched comparison, the rate of severe complications (Clavien Dindo grade 3 or higher) was lower in the PREPARE group, but this difference lost significance in the matched comparison (Table 2; unmatched analysis 24% vs 46%, *p* = 0.015; matched analysis 32% vs 47%, *p* = 0.159).

On multivariate regression analysis of the whole cohort, participation in PREPARE was associated with a 77% lower incidence of post-operative pneumonia (Table 3; OR 0.23 95% CI 0.09 to 0.55 *p* = 0.001). There was no significant effect of age, the use of neo-adjuvant treatment, pre-operative stage, CCI, or ASA grade.

**Table 3:** Factors associated with development of post-operative pneumonia

Variable	Odds ratio (95% CI)	SE	*p* value
Age	1.04 (0.97 to 1.12)	0.04	0.280
ASA grade	1.19 (0.39 to 3.66)	0.57	0.761
Clinical stage (II)	3.39 (0.37 to 31.28)	1.13	0.281
Clinical stage (III)	0.72 (0.12 to 4.00)	0.88	0.707
Clinical stage (IV)	1.94 (0.60 to 6.31)	0.60	0.271
CCI	0.92 (0.49 to 1.73)	0.32	0.806
NAC	1.34 (0.22 to 8.02)	0.91	0.751
Prehabilitation	0.23 (0.09 to 0.55)	0.46	0.001

*CCI*, Charlson Comorbidity Index; *NAC*, neoadjuvant chemotherapy; *ASA*, American Society of Anesthesiologist physical status classification

There was a non-significant trend for a higher 30-day readmission rate in the PREPARE patients (Table 2). In the overall PREPARE cohort, 13 patients were readmitted within 30 days (18%). The reasons for readmission included pneumonia (*n* = 2); recurrent pleural effusions (*n* = 2); pneumothorax (*n* = 1); wound infection (*n* = 2); inadequate pain control (*n* = 2); cardiac arrythmia (*n* = 1); diarrhoea (*n* = 1); intra-abdominal collection (*n* = 1); and a new diagnosis of metastatic disease (*n* = 1). The median length of stay for the readmissions was 6 days (IQR 3 to 13 days).

### Compliance with Enhanced Recovery Protocol

There was a higher rate of compliance with the post-operative target for removal of nasogastric (NG) tubes in the PREPARE patients (Table 2). In the unmatched analysis, there was also a significantly higher compliance with the removal of surgical drains in the PREPARE patients, but this lost significance once propensity score matching was performed. Compliance with all other ERP elements was comparable between the two groups.

## Discussion

This study has found a significantly lower incidence of post-operative pneumonia and a shorter length of stay in patients who have undergone the PREPARE for Surgery programme in comparison to controls. We also observed a trend for a lower overall incidence of complications in the PREPARE cohort, although this did not reach statistical significance.

To date, this is the first study that has shown improved post-operative outcomes in oesophageal cancer patients who have undergone prehabilitation. Two meta-analyses into prehabilitation in patients undergoing intra-abdominal operations have reported 37% and 41% reductions in post-operative complications.^22^,^27^ The impact of prehabilitation on respiratory complications is particularly notable, with a 60% lower incidence in comparison to controls.^27^ In this study, we observed that prehabilitation was associated with a 77% reduction in the incidence of pneumonia following oesophagectomy.

A previous study of patients participating in the PREPARE for Surgery programme found that the incidence of post-operative pneumonia was significantly related to the amount of exercise completed in the pre-operative period.^32^ The mechanism by which prehabilitation reduces post-operative pneumonia is likely to be multi-factorial. Prehabilitation has been shown to improve cardio-respiratory fitness in a range of patient populations, including oesophageal cancer patients.^16^,^17^,^23^,^31^,^42^. Oesophageal cancer patients have a high incidence of sarcopenia,^43^,^44^ which is associated with post-operative respiratory complications,^45^,^46^ and further research is needed to assess the impact of prehabilitation on body composition. Other possible mechanisms include improving respiratory physiology with stronger respiratory muscle function and better clearance of secretions. Some prehabilitation programmes include specific inspiratory muscle training,^15^,^16^,^20^ although this is not a component of the PREPARE programme.

Reported rates of complications after an oesophagectomy often exceed 60%^5^,^47^ and respiratory complications are particularly commonplace, with previous studies reporting post-operative pneumonia rates of between 16 and 50%.^2^,^6^,^48^,^49^ Within this study, all patients underwent an open thoracotomy and approximately 90% of patients underwent an open laparotomy for the abdominal stage (Table 1). This approach is used with the aim of maximising oncological outcomes but is known to be associated with a greater incidence of post-operative pneumonia.^50^ The high incidence of post-operative pneumonia was a driving factor behind the adoption of prehabilitation.

Exercise during chemotherapy is challenging.^51^ We found adherence to the personalised exercise prescriptions was lower whilst patients were undergoing neoadjuvant chemotherapy in comparison to once it was completed (55% and 66% respectively). The exercises were personalised and adapted over the course of each patient’s programme dependent on their progress and clinical condition. This dynamic approach was adopted with the aim of maximising participation. Whilst the adherence percentages show that most patients did not fully complete their exercises as prescribed, it is notable that no patients asked to withdraw from the programme and the average amount of physical activity completed in MET minutes week^−1^ exceeded the WHO recommendations both during and after chemotherapy.^33^

Compliance with six out of the seven ERP elements in the matched analysis were comparable between the two groups, suggesting that there were no substantial differences in post-operative care to account for the lower rate of pneumonia and shorter hospital stay. However, in the prehabilitation group, there was a higher rate of compliance with the target to remove NG tubes by day 5 after surgery (Fig. 1 and Table 2).

As the majority of controls patients were from a historical cohort, this could reflect a small change in practice around NG tube removal. However, the relationship between ERP compliance and complications is complex; complications may result from poor compliance but complications themselves will also cause deviation from the protocol and lower compliance.^52^ For example, better compliance with the target for NG tube removal in the PREPARE group may occur because of the lower incidence of post-operative pneumonia. In our practice, an elevation in inflammatory markers raises concerns about an anastomotic leak. Therefore, a rise in inflammatory markers from pneumonia may delay NG tube removal until an anastomotic leak has been excluded by other investigations, and this could explain the lower compliance with the target for NG tube removal in the control group.

The readmission rate in our prehabilitation patients was 18% in the unmatched comparison and 21% in the matched analysis. These figures are higher than recent benchmarking outcome data which reported an 11% readmission rate.^5^ In the whole PREPARE cohort, nine of the 13 readmissions were due to post-operative complications and four were due to symptom control or disease progression. These complications were included in the comparison of 60-day complications between the two study groups. Although the overall rate of pulmonary complications was lower in the PREPARE group, the incidence of post-operative complications after an oesophagectomy is still high.^5^,^6^ The PREPARE cohort had a shorter length of stay and therefore complications that may otherwise have been managed during the index admission occurred in the community setting.

Identifying ways to reduce readmission and provide more support following discharge should be a focus of future research. Ambulatory care for symptom control and low-grade complications, such as wound infections, could be employed to prevent or limit readmission, which itself may serve to further delay post-operative functional recovery. The ethos of prehabilitation could be extended to recovery at home after surgery, with structured exercise and psychological interventions to facilitate supported self-management.

There are several limitations to this study. Firstly, it is an observational study and the intervention was not randomised between the two arms of this study. To reduce the impact of confounding factors between the two groups, a propensity score matched analysis was performed. Although this reduced the size of the study population, the significant differences in the incidence of pneumonia and length of stay remained. The number of cases and controls included in this study limited the number of variables that could be added to the propensity score. The number of controls was limited by the availability of data. It was not possible to add cases from before 2015 as a new enhanced recovery protocol was implemented at this point and therefore significant differences occurred in other aspects of peri-operative care independent to the use of prehabilitation.

Measurements of baseline fitness and physical activity were not recorded for the control patients so this could not be added to the propensity score. Whilst there was no difference in comorbidities between the two groups (with the exception of a higher incidence of respiratory comorbidities in the PREPARE group), we were unable to compare the two cohorts for cardio-respiratory fitness and lung function, which could have impacted on post-operative pneumonia. Lung function tests were performed only when indicated and cardio-pulmonary exercise testing was not routinely performed. Sub-maximal exercise testing was performed only in the prehabilitation group and thus was not available for the controls. Psychometric variables, such as anxiety or motivation, which may influence post-operative recovery were also not recorded for the control patients. However, there was no significant difference in known psychiatric comorbidities between the two groups (Table 1).

Further research is needed into the effects of prehabilitation on long-term outcomes and recovery. We have not reported an economic analysis of prehabilitation, and this will form an area of future research. The PREPARE for Surgery programme was developed and implemented using quality improvement methodology, and was co-designed by patients, carers, and healthcare professionals. Given the growing evidence showing significant benefits from prehabilitation, a study into the barriers and facilitating factors for the delivery of prehabilitation should be undertaken to aid the roll-out of prehabilitation to a wider range of clinical settings.

## Conclusion

Patients undergoing an oesophagectomy are at high-risk of respiratory complications. Using a propensity score matched analysis, we have observed a lower rate of post-operative pneumonia and shorter length of stay in oesophageal cancer patients who have undergone home-based, multimodal prehabilitation. This model of home-based prehabilitation is especially important in centralised cancer specialties such as oesophageal cancer. Success hinges not on the location of the intervention, but the level of personalisation, monitoring, and support provided by the clinical team.

## References

[1] Allum WH, Blazeby JM, Griffin SM, Cunningham D, Jankowski JA, Wong R (2011). Guidelines for the management of oesophageal and gastric cancer. Gut.

[2] Chadwick G, Varagunam M, Groene O, Cromwell D, Hardwick R, Maynard N, Riley S, Crosby T, Greenaway K (2015). National Oesophago-Gastric Cancer Audit 2015.

[3] Backemar L, Lagergren P, Johar A, Lagergren J (2015). Impact of co- morbidity on mortality after oesophageal cancer surgery. Br J Surg.

[4] McIsaac DI, Bryson GL, van Walraven C (2016). Association of Frailty and 1- Year Postoperative Mortality Following Major Elective Noncardiac Surgery: A Population-Based Cohort Study. JAMA Surg.

[5] Low DE, Kuppusamy MK, Alderson D, Cecconello I, Chang AC, Darling G, Davies AD, Journo XB, Gisbertz SS, Griffin SM, Hardwick R, Hoelscher A, Hofstetter W, Jobe B, Kitagawa Y, Law S, Mariettte C, Maynard N, Morse CR, Nafteux P, Pera M, Pramesh CS, Puig S, Reynolds JV, Schroeder W, Smithers M, Wijnhoven BPL (2019). Benchmarking Complications Associated with Esophagectomy. Ann Surg.

[6] Paul S, Altorki N (2014). Outcomes in the management of esophageal cancer. J Surg Oncol.

[7] Doorakkers E, Konings P, Mattsson F, Lagergren J, Brusselaers N. Early Complications Following Oesophagectomy for Cancer in Relation to Long- Term Healthcare Utilisation: A Prospective Population-Based Cohort Study (Oesophageal Cancer and Healthcare- Utilisation). PLoS ONE 2015; 10(3) 10.1371/journal.pone.0121080 10.1371/journal.pone.0121080 PMC4358940 25768921

[8] Rutegård M, Lagergren P, Rouvelas I, Mason R, Lagergren J (2012). Surgical complications and long- term survival after esophagectomy for cancer in a nationwide Swedish cohort study. Eur J Surg Oncol.

[9] Wynter-Blyth V, Moorthy K. Prehabilitation: preparing patients for surgery. BMJ 2017; 358 10.1136/bmj.j3702 10.1136/bmj.j3702 28790033

[10] Carli F, Silver JK, Feldman LS, McKee A, Gilman S, Gillis C, Scheede-Bergdahl C, Gamsa A, Stout N, Hirsch B (2017). Surgical Prehabilitation in Patients with Cancer: State-of-the-science and recommendations for future research from a panel of subject matter experts. Phys Med Rehabil Clin N Am.

[11] Desborough JP (2000). The stress response to trauma and surgery. Br J Anaesth.

[12] Hoppe S, Rainfray M, Fonck M, Hoppenreys L, Blanc J, Ceccaldi J, Mertens C, Blanc-Bisson C, Imbert Y, Cany L, Vogt L, Dauba J, Houede N, Bellera CA, Floquet A, Fabry MN, Ravaud A, Chakiba C, Mathoulin-Pelissier S, Soubeyran P (2013). Functional decline in older patients with cancer receiving first- line chemotherapy. J Clin Oncol.

[13] Jack S, West MA, Raw D, Marwood S, Ambler G, Cope TM, Shrotri M, Sturgess RP, Calverley PMA, Ottensmeier CH, Grocott MPW (2014). The effect of neoadjuvant chemotherapy on physical fitness and survival in patients undergoing oesophagogastric cancer surgery. Eur J Surg Oncol.

[14] West MA, Loughney L, Lythgoe D, Barben CP, Sripadam R, Kemp GJ, Grocott MP, Jack S (2015). Effect of prehabilitation on objectively measured physical fitness after neoadjuvant treatment in preoperative rectal cancer patients: a blinded interventional pilot study. Br J Anaesth.

[15] Levett DZH, Edwards M, Grocott M, Mythen M (2016). Preparing the patient for surgery to improve outcomes. Best Pract Res Clin Anaesthesiol.

[16] Piraux E, Caty G, Reychler G (2018). Effects of preoperative combined aerobic and resistance exercise training in cancer patients undergoing tumour resection surgery: A systematic review of randomised trials. Surg Oncol.

[17] Vermillion SA, James A, Dorrell RD, Brubaker P, Mihalko SL, Hill AR, Clark CJ (2018). Preoperative exercise therapy for gastrointestinal cancer patients: a systematic review. Syst Rev.

[18] Moorthy K, Wynter-Blyth V (2017). Prehabilitation in perioperative care. Br J Surg.

[19] Minnella EM, Carli F (2018). Prehabilitation and functional recovery for colorectal cancer patients. Eur J Surg Oncol.

[20] Zylstra J, Boshier P, Whyte GP, Low DE, Davies AR (2018). Peri-operative patient optimization for oesophageal cancer surgery – From prehabilitation to enhanced recovery. Best Pract Res Clin Gastroenterol.

[21] Santa Mina D, Clarke H, Ritvo P, Leung YW, Matthew AG, Katz J, Trachtenberg J, Alibhai SMH (2014). Effect of total-body prehabilitation on postoperative outcomes: a systematic review and meta-analysis. Physiotherapy.

[22] Moran J, Guinan E, McCormick P, Larkin J, Mockler D, Hussey J, Moriarty J, Wilson F (2016). The ability of prehabilitation to influence postoperative outcome after intra- abdominal operation: A systematic review and meta-analysis. Surgery.

[23] Minnella EM, Bousquet-Dion G, Awasthi R, Scheede-Bergdahl C, Carli F (2017). Multimodal prehabilitation improves functional capacity before and after colorectal surgery for cancer: a five-year research experience. Acta Oncol.

[24] Lau CSM, Chamberlain RS. Prehabilitation Programs Improve Exercise Capacity Before and After Surgery in Gastrointestinal Cancer Surgery Patients: A Meta-Analysis. J Gastrointest Surg 2019 10.1007/s11605-019-04436-1 10.1007/s11605-019-04436-1 31768827

[25] Santa Mina D, Hilton WJ, Matthew AG, Awasthi R, Bousquet-Dion G, Alibhai SMH, Au D, Fleshner NE, Finelli A, Clarke H, Aprikian A, Tanguay S, Carli F (2018). Prehabilitation for radical prostatectomy: A multicentre randomized controlled trial. Surg Oncol.

[26] Minnella EM, Awasthi R, Bousquet-Dion G, Ferreira V, Austin B, Audi C, Tanguay S, Aprikian A, Carli F, Kassouf W. Multimodal Prehabilitation to Enhance Functional Capacity Following Radical Cystectomy: A Randomized Controlled Trial. Eur Urol Focus 2019 10.1016/j.euf.2019.05. 10.1016/j.euf.2019.05.016 31186173

[27] Hughes M, Hackney R, Lamb P, Wigmore S, Deans C, Skipworth R (2019). Prehabilitation before Major Abdominal Surgery: A Systematic Review and Meta-Analysis. World J Surg.

[28] Barbour AP, Lagergren P, Hughes R, Alderson D, Barham CP, Blazeby JM (2008). Health- related quality of life among patients with adenocarcinoma of the gastro- oesophageal junction treated by gastrectomy or oesophagectomy. Br J Surg.

[29] Akkerman RDL, Haverkamp L, van Rossum PSN, van Hillegersberg R, Ruurda JP (2015). Long- term quality of life after oesophagectomy with gastric conduit interposition for cancer. Eur J Cancer.

[30] Dewberry LC, Wingrove LJ, Marsh MD, Glode AE, Schefter TE, Leong S, Purcell WT, McCarter MD (2019). Pilot Prehabilitation Program for Patients with Esophageal Cancer During Neoadjuvant Therapy and Surgery. J Surg Res.

[31] Minnella EM, Awasthi R, Loiselle S, Agnihotram RV, Ferri LE, Carli F (2018). Effect of Exercise and Nutrition Prehabilitation on Functional Capacity in Esophagogastric Cancer Surgery: A Randomized Clinical Trial. JAMA Surg.

[32] Halliday LJ, Doganay E, Wynter-Blyth V, Osborn H, Buckley J, Moorthy K. Adherence to Pre-operative Exercise and the Response to Prehabilitation in Oesophageal Cancer Patients. J Gastrointest Surg 2020 10.1007/s11605-020-04561-2 10.1007/s11605-020-04561-2 PMC8007503 32314231

[33] World Health Organisation. Global recommendations on physical activity for health. Available from : https://apps.who.int/iris/bitstream/handle/10665/44399/9789241599979_eng.pdf?sequence=1 [Accessed November 10, 2018]

[34] American College of Sports Medicine (2010). ACSMs guidelines for exercise testing and prescription.

[35] Colberg SR, Swain DP, Vinik AI (2003). Use of heart rate reserve and rating of perceived exertion to prescribe exercise intensity in diabetic autonomic neuropathy. Diabetes Care.

[36] Borg G (1998). Borg’s Perceived Exertion and Pain Scales.

[37] Buckley J, Jones J. Tables for assessing, monitoring and guiding physical activity/exercise intensity in programmes for cardiovascular disease prevention and rehabilitation. London: British Association for Cardiovascular Prevention and Rehabilitation; 2012.

[38] Rollnick S, Butler CC, Kinnersley P, Gregory J, Mash B. Motivational interviewing. BMJ 2010; 340(7758) 10.1136/bmj.c1900 10.1136/bmj.c1900 20423957

[39] Low DE, Alderson D, Cecconello I, Chang AC, Darling GE, DʼJourno XB, Griffin SM, Hölscher AH, Hofstetter WL, Jobe BA, Kitagawa Y, Kucharczuk JC, Law SYK, Lerut TE, Maynard N, Pera M, Peters JH, Pramesh CS, Reynolds JV, Smithers BM, van Lanschot JJB. International Consensus on Standardization of Data Collection for Complications Associated With Esophagectomy: Esophagectomy Complications Consensus Group (ECCG). Ann Surg 2015; 262(2): 286-294 10.1097/SLA.0000000000001098 25607756

[40] Streiner DL, Norman GR (2012). The Pros and Cons of Propensity Scores. Chest.

[41] D’ Agostino RB (1998). Propensity score methods for bias reduction in the comparison of a treatment to a non-randomized control group. Stat Med.

[42] West MA, Astin R, Moyses HE, Cave J, White D, Levett DZH (2019). Exercise prehabilitation may lead to augmented tumor regression following neoadjuvant chemoradiotherapy in locally advanced rectal cancer. Acta Oncol.

[43] Elliott JA, Doyle SL, Murphy CF, King S, Guinan EM, Beddy P, Ravi N, Reynolds JV (2017). Sarcopenia: Prevalence, and Impact on Operative and Oncologic Outcomes in the Multimodal Management of Locally Advanced Esophageal Cancer. Ann Surg.

[44] Yip C, Goh V, Davies A, Gossage J, Mitchell-Hay R, Hynes O, Maisey N, Ross P, Gaya A, Landau DB, Cook GJ, Griffin N, Mason R (2014). Assessment of sarcopenia and changes in body composition after neoadjuvant chemotherapy and associations with clinical outcomes in oesophageal cancer. Eur Radiol.

[45] Boshier PR, Heneghan R, Markar SR, Baracos VE, Low DE. Assessment of body composition and sarcopenia in patients with esophageal cancer: a systematic review and meta-analysis. Dis Esophagus 2018; 31(8) 10.1093/dote/doy047 10.1093/dote/doy047 29846548

[46] Makiura D, Ono R, Inoue J, Kashiwa M, Oshikiri T, Nakamura T, Kakeji Y, Sakai Y, Miura Y (2016). Preoperative sarcopenia is a predictor of postoperative pulmonary complications in esophageal cancer following esophagectomy: A retrospective cohort study. Journal of Geriatric Oncology.

[47] Sinclair RCF, Phillips AW, Navidi M, Griffin SM, Snowden CP (2017). Pre-operative variables including fitness associated with complications after oesophagectomy. Anaesthesia.

[48] Varagunam M, Park MH, Sinha S, Cromwell D, Maynard N, Crosby T, Trudgill N, Michalowski J, Salvador A, Napper R (2019). National Oesophago-Gastric Cancer Audit 2018.

[49] Goense L, van Rossum PSN, Tromp M, Joore HC, van Dijk D, Kroese AC, Ruurda JP, van Hillegersberg R. Intraoperative and postoperative risk factors for anastomotic leakage and pneumonia after esophagectomy for cancer. Dis Esophagus 2017; 30. Available from: 10.1111/dote.12517 10.1111/dote.12517 27353216

[50] Xiong W, Li R, Lei H, Jiang Z (2017). Comparison of outcomes between minimally invasive oesophagectomy and open oesophagectomy for oesophageal cancer. ANZ J Surg.

[51] Lahart IM, Metsios GS, Nevill AM, Carmichael AR (2014). Physical activity levels in women attending breast screening, receiving chemotherapy and post-breast cancer treatment; a cross-sectional study. Int J Environ Res Public Health.

[52] Thorn C, White I, Burch J, Malietzis G, Kennedy R, Jenkins J (2016). Active and passive compliance in an enhanced recovery programme. Int J Colorectal Dis.

